# Consumer satisfaction-oriented emotional marketing in foreign trade

**DOI:** 10.3389/fpsyg.2022.960042

**Published:** 2022-08-25

**Authors:** Lei Zhu, Yanhua Gao, Weijing Chen, Hao Ren

**Affiliations:** ^1^School of Public Administration, Xi'an University of Architecture and Technology, Xi'an, China; ^2^Graduate School of Management, Management and Science University, Shah Alam, Malaysia; ^3^School of Business Administration, Xi'an Eurasia University, Xi'an, China; ^4^School of Humanities and Education, Xijing University, Xi'an, China

**Keywords:** customers, competitors, departmental responsiveness, customer satisfaction, foreign trade

## Abstract

This research uses an experimental approach to investigate the relationship between market orientation of a company and its level of success in international business. The aim of the study was to develop and use a market orientation scale that is appropriate to the sector. It was discovered that there are four hidden traits that drive market orientation. These include customers, rivals, departmental response, and overall customer satisfaction. According to the results, orientation toward one's customers is more essential than any of the other traits, while orientation toward one's competitors has an inverted *U*-shaped connection with performance. The performance of the firm was not found to correlate in any way with the responsiveness of its departments. With the help of the comprehensive conceptualization, managers are able to develop specific kinds of orientations that are essential for higher levels of performance.

## Introduction

At the time of writing this article, surroundings of most companies were characterized by increasing competition and environmental concerns. This harsh reality has forced most firms to create ways for dealing with it or risk extinction. Many business techniques and methods for success in today's dynamic business environment have been proposed by managers as a consequence of their intense efforts to get a competitive edge (Day and Wensley, 1988). Marketers and scholars are interested in marketing philosophy in this context since marketing is considered only as an operational and strategic issue for corporations. As a result, current empirical investigations tend to be sector-specific, while earlier research aimed to advance to theory testing and evaluation of the universality of an idea (Chee and Peng, 1996). In the vast majority of studies, the association between market orientation and organizational has been shown to be positive. A study of impacts of market orientation should be considered since managers must know the factors required for developing a market culture.

Among the numerous contributions this study will make to the field of market orientation is the following: Following development and testing of the specialized market-oriented scale of the machine tool industry, we focused on underlying issues of market performance. First, theoretical consideration was given to the degree to which business performance elements were related to performance; second, empirical testing was omitted in order to avoid the approach by focusing on new assessments of market orientation. Finally, using a variety of financial and operational performance indicators, performance metrics are derived from a holistic view of the firm. The market orientation of the vehicle manufacturing sector is discussed in this section, along with an explanation of how it might affect the performance of a company.

This same process was used to create either the sample frame or the data collection methods, which are described later in the article. The applicability of the new market orientation scale is investigated using a variety of statistical methods. Using multiple linear regression or ANOVA findings, other variables supporting market orientation on company performance are investigated. Following that, the study results are reviewed in relation to past study results. The findings and goals of this study are presented at the conclusion of the article for the benefit of machine tool executives and practitioners.

Researchers have mostly concentrated on service firms including those that operate across sectors in this area of study. But there seemed to be no empirical study in the British machine tool sector on the link between market orientation and performance, and an examination is being conducted. For another reason, the machine tool sector is considered a positive indicator tone for the entire manufacturing industry, which relies on new and innovative equipment from it. There have been few attempts to tailor their constructs to a single sector spite of the importance in relationship marketing and recent advances in quantification.

This study aims to build an industry-specific market orientation domain by integrating three popular market orientation notions (Deng and Dart, 1994). This kind of fusion may be useful for empirical studies in future. The design was applied to the car manufacturing industry, and operational adjustments were investigated in order to carry the study forward.

A cross-sectional study by Diamantopoulos and Hart (1993) was carried out in the United Kingdom. For this study, a sample of consumer items, consumer services, and industrial goods and services was gathered. When research is not differentiated, it is harder to decipher the impacts of environmental variables such as technical advancement and market growth. All participants in the machine tool industry are affected by the same environmental variables because of the exclusive emphasis of the study on this particular industry. In the machine tool industry, small- and medium-sized firms (SMEs) manufacture a wide variety of product types and sizes (Rhoads and Thorn, 1996). Non-CNC machine tool makers are in a different industry segment in this business (Deighton and Henderson, 1994).

A dynamic corporate environment necessitates ongoing product innovation or quality improvement to meet customer needs. Success of a company depends on launching new products in order to match customer demand. For marketing objectives, market segmentation is vital. New products should be positioned in such a way that they appeal to their intended audience. After that, the degree of product quality satisfaction that is ideal for the target audience should be determined. Technology (NPD) is widely used by business units to design a product that fulfills the desired customers' satisfaction while keeping within budget or consuming the minimum of resources.

The QFD method has been utilized by practitioners since the 1970's (Cohen, 1995) to build new products or improve the existing ones. Decision-making, production, service, and education have all benefited from the use of QFD (Chan and Wu, 2002). QFD provides a systematic approach to convert customer demands into technical requirements. Several academics have used QFD to provide quantitative answers to a variety of problems. Using mathematical programming, quantitative approaches are widely used to develop models to determine the optimum criteria for design to maximize customer satisfaction while staying within budget, time, and/or resource restrictions (**?**Kim, 1997).

The aim of every company is to maintain a long-term relationship with customers and their business organization. In order to acquire the potential customers, needs and demands should be acknowledged; also, customer satisfaction has a great impact on the entire business operations. Therefore, it is very important to the organization to understand what exactly the customers need and how to gain loyalty for the successful business. As it is discussed in the following section, the customer plays a crucial role in the market chain process. To make it clearer, satisfied customers are the ones who creates the possibility of new customers. If the existing customers are satisfied with the product and service, then there are chances of recommendation to new ones. This will lead to the increasing number of customers and could maintain the level of the relationship with the customers. This thesis has been studied in-depth to understand the correlation between customer satisfaction and customer loyalty. The authors realized that the customer plays a crucial role in customer satisfaction and customer loyalty and is the root of the success. The authors figured out if the customers are satisfied with the quality of the service and perform the tasks according to the customer's demand. The company is satisfied customers along with loyalty. It can be said that customer satisfaction is the key component of business profitability because once the customer reaches their satisfaction level, it may influence them to consume the service continuously. Moreover, they share their experience with other people, which creates the possibility of new customers. Likewise, dissatisfied people also give their opinion about the products and about their unfortunate experience, which leads toward a decline in the number of customers.

## Methods

For the most part, HOQ information is pooled in order to determine the importance of DRs and even the degree to which they have succeeded in meeting their objectives. The normalization approach is extensively used by QFD academics and practitioners (**?**). Wasserman's normalization has been widely utilized. This model integrates DR correlations, based on Lyman's normalization concept (Lyman, 1990), as follows:


(1) $$\begin{array}{l} R_{ij}^{\prime }=\frac{\sum_{k=1}^{n}R_{ik}.\gamma_{kj}}{\sum_{j=1}^{n}\overset{n}{\underset{k=1}{\sum }}R_{ik}.\gamma_{kj}} \end{array}$$

**Figure 1:**
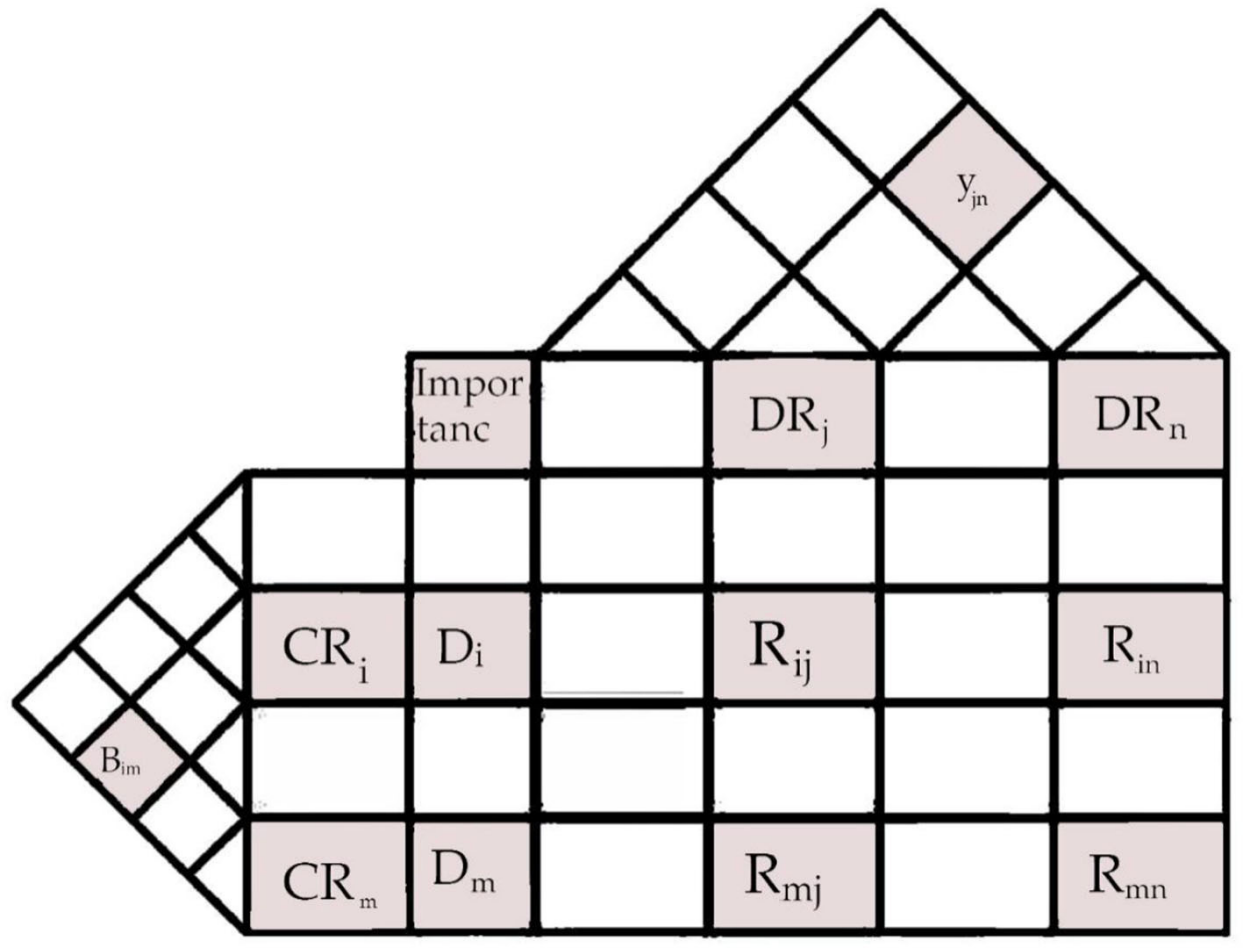
General HOQ structure.

From the idea of vector space, where γ_*kj*_ signifies the technical relationship between *DR*_*k*_ and *CR*_*j*_. *R*_*ik*_ relational intensity between *DR*_*k*_ and *CR*_*j*_ is quantified using a three-point scale to describe the weak, moderate, and strong connection in the preceding equation. Despite its widespread use, Wasserman's normalization methodology has numerous flaws. It presupposes, for example, that customer needs are mutually exclusive. However, in reality, this may not be the case. More crucially, this model may provide a relational intensity for a pair of CRs and DRs that were not included in the original design data. As a result, the model may yield irrational results. Chen and Chen (2014) presented the following updated normalization model in response to these flaws in Wasserman's normalization model:


(2) $$\begin{array}{l} R_{ij}^{norm}=\frac{\left(\sum_{k=1}^{m}\gamma_{kj}\right)R_{ij}}{\sum_{i=1}^{m}\left(\overset{m}{\underset{k=1}{\sum }}\gamma_{kj}\right)R_{ij}} \end{array}$$

where $R_{ij}^{norm}$ the normalized connection between *CR*_*i*_ and *DR*_*j*_ is denoted by the inappropriate outputs from Wasserman's model may be avoided by using the following updated normalization approach to incorporate design knowledge. In addition, Chen and Chen (2014) developed the following normalization model to integrate CRs, similar to that in Equation 2, to find the normalized weights of CRs, taking into account the likelihood that certain CRs are linked, as shown in Figure 1.


(3) $$\begin{array}{l} d_{i}^{norm}=\frac{\left(\sum_{l=1}^{m}\beta_{il}\right)d_{i}}{\sum_{i=1}^{m}\left(\overset{m}{\underset{l=1}{\sum }}\beta_{il}\right)d_{i}} \end{array}$$

### Quality level and satisfaction function

**Figure 2:**
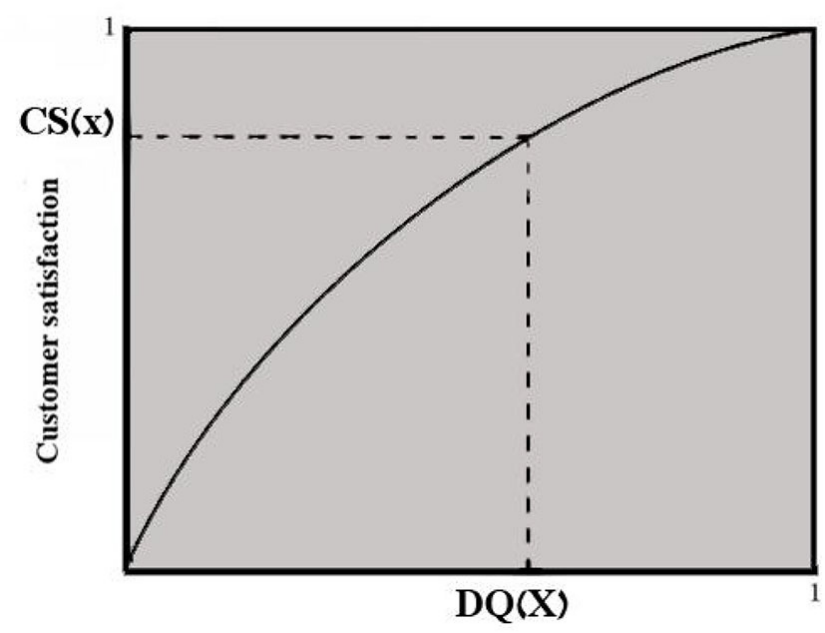
Customer satisfaction (CS) and design quality functional curve (DQ).

It is the fundamental objective of product development and improvement to meet or exceed customers' needs or improve customer satisfaction. Customer happiness is an important goal for something like the target market (Erevelles and Leavitt, 1992). Customer pleasure is among the most significant customer constructs (Morgan et al., 1996). Customer happiness is vital in marketing, but it also has a significant impact on consumer purchasing behavior. According to Anderson and Sullivan (Anderson and Sullivan, 1993), customer pleasure may be defined as a relationship between perceived quality of consumers and an adjustment amount to represent the customers' view of confirmation/disconfirmation. Their investigation demonstrates a concave down feature of perceived quality. In the current study, a sloping down function is utilized to hypothesize that the excellence meant for such product throughout the design stage may be observed by target customers in the market place, that is, any creative quality standard has a positive relationship with perceived service quality. Figure 2 displays the link between customer satisfaction (CS) and quality attributes as a concave down function or a straight line (DQ).

Customer happiness may be represented as a function of the DR fulfillment levels in QFD operations. This is the cost necessary to fulfill the technical requirement for DR and fully satisfy the client. X is a decision variable that indicates the quality level of DR required to fully satisfy the customer. Assume for simplicity that the cost necessary to attain DR, x, is CX, that is, the required cost is proportionate to the fulfillment level. If xj is 0, then the cost of DR is 0. Using the whole budget C for DR will also accomplish the complete technical requirement, xj, and full customer satisfaction.

Let *X* = [*x*_1_, *x*_2_, ...., *x*_*n*_] be the vector carrying the degrees of technical requirement fulfillment for all DRs. The overall DQ of a new product is calculated by taking the average of all DR satisfaction levels.


(4) $$\begin{array}{l} DQ\left(X\right)=\frac{1}{n}\overset{n}{\underset{j=1}{\sum }}x_{j},0\leq x_{j}\leq 1 \end{array}$$

In which 0 ≤ *DQ*(*X*) ≤ 1. In other words, *x*_*j*_ represents the level of design excellence. Furthermore, based on earlier research (e.g., **?**Kim, 1997), customer satisfaction may be expressed as utilizing the normalized information from the HOQ, $R_{ij}^{norm}$ and $d_{i}^{norm}$ in Equations 2, 3, respectively.


(5) $$\begin{array}{l} \frac{1}{n}\overset{n}{\underset{j=1}{\sum }}x_{j}\geq {\left(\sum_{i=1}^{m}d_{i}^{norm}\sum_{j=1}^{m}R_{ij}^{norm}.x_{j}\right)}^{K} \end{array}$$

**Figure 3:**
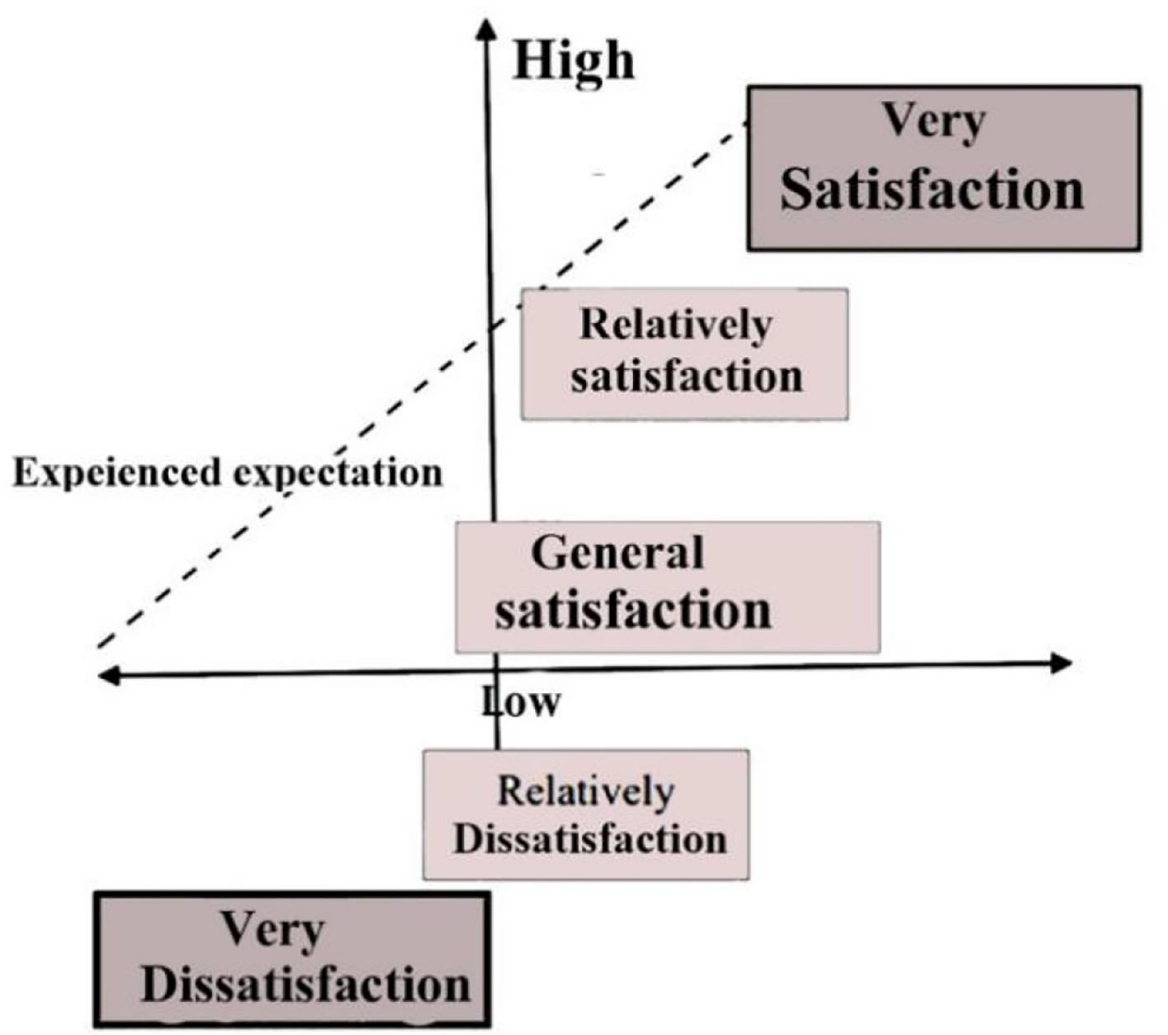
Customer satisfaction.

According to Anderson and Sullivan's study, customer satisfaction is described as a concave down relationship of a product design quality for a certain market group (Anderson and Sullivan, 1993). A function like this is shown in Figure 3. The decreasing marginal impact is represented by the concave down curve; as design quality improves, the marginal effect of customer satisfaction decreases. The following simple equation is used to show this:


(6) $$\begin{array}{l} CS\;(X)=\sqrt[{k}]{DQ},k>1 \end{array}$$

where *k* > 1 is a distinguishing variable that represents a market segment's customer preference for a product design quality level. The Equation 6, as previously indicated, explains features of the link between and. In practice, if the intended customer satisfaction level is determined as meeting the target market, Equation 6 is utilized to transform the intended value into the matched value according to the required design quality level.

### Proposed non-linear programming model


(7) $$\begin{array}{l} Min\overset{n}{\underset{j=1}{\sum }}C_{j}x_{j} \end{array}$$

Subject to


(8) $$\begin{array}{l} \sum_{i=1}^{n}d_{i}^{norm}\sum_{j=1}^{n}R_{ij}^{norm},x_{j}\geq \delta ,0\leq \delta \leq 1 \end{array}$$

(9) $$\begin{array}{l} \sum_{i=1}^{n}d_{i}^{norm}\sum_{j=1}^{n}R_{ij}^{norm},x_{j}\geq \delta ,0\leq \delta \leq 1 \end{array}$$

(10) $$\begin{array}{l} \frac{1}{n}\overset{n}{\underset{j=1}{\sum }}x_{j}-{\left(\sum_{i=1}^{n}d_{i}^{norm}\sum_{j=1}^{n}R_{ij}^{norm}.x_{j}\right)}^{k}\geq 0,k>1 \end{array}$$

(11) $$\begin{array}{l} \overset{n}{\underset{j=1}{\sum }}x_{j}\geq \;n\;\alpha \end{array}$$

(12) $$\begin{array}{l} \alpha =\sigma^{k} \end{array}$$

(13) $$\begin{array}{l} \sum_{j=1}^{m}R_{ij}^{norm}.x_{j}\geq \varepsilon_{i} \end{array}$$

(14) $$\begin{array}{l} \varepsilon_{i}=t_{1}d_{i}^{norm},t_{1}>0 \end{array}$$

(15) $$\begin{array}{l} \rho_{j}\leq x_{j}\leq 1,0\leq \rho_{j}\leq 1,j=1,...,n \end{array}$$

(16) $$\begin{array}{l} \rho_{j},=t_{2}\left(\sum_{i=1}^{m}d_{i}^{norm}.R_{ij}^{norm}\right)\alpha \end{array}$$

Note that constraint may be shown in the form of an object as follows:


(17) $$\begin{array}{l} \overset{n}{\underset{j=1}{\sum }}\left(\sum_{i=1}^{m}d_{i}^{norm}.R_{ij}^{norm}\right).x_{j}\geq \delta . \end{array}$$

If $w_{j}^{norm}$ is the normalized significance of DRj to the contribution of all CRs,


(18) $$\begin{array}{l} w_{j}^{norm}=\sum_{i=1}^{m}d_{i}^{norm}.R_{ij}^{norm} \end{array}$$

(19) $$\begin{array}{l} \overset{n}{\underset{j=1}{\sum }}w_{j}^{norm}.x_{j}\geq \delta \end{array}$$

Finally,


(20) $$\begin{array}{l} \rho_{j}=t_{2}w_{j}^{norm}\alpha \end{array}$$

### Customer satisfaction

The happiness of a customer is both transitory and variable. Only by emphasizing “customer centricity” can businesses hope to keep their customers happy and loyal. As a result, if competitors enhance customer experience, corporate customers might be at risk. Enhancing customer happiness requires taking the expectations of the client into account. The quality of the service, the quality of the product, and the overall value for the money all play a role in customer satisfaction. In order to have happy customers, the company must first have happy employees. Staff may have a huge impact on customer satisfaction when they have a positive impact (Hill et al., 2007).

The pursuit of happiness is a fluid goal that is subject to shifts in light of changing circumstances. The degree to which one is satisfied with a product or service may vary widely during the course of its usage or experience, in particular. Customer satisfaction is influenced by product or service quality and quality assessments (Lovelock and Wright, 2007).

Emotional marketing is a way to connect with your consumers, develop meaningful relationships, and cultivate lasting customers. An extension of that is emotional branding, the art of storytelling that helps connect a product or service with an appropriate audience. Customer emotional reactions, attitudes, and their impression of equality all impact satisfaction (Zeithaml and Bitner, 2003). Once a company knows its target audience on a personal and an emotional level, this knowledge should be used to form a story that they can relate to. People love to hear stories that they can empathize with, learn from, or be inspired by. A company should offer something that makes readers feel excited to share with their peers. Customer loyalty, product longevity, and good word-of-mouth advertising may all be attributed to efforts of a company to improve customer happiness and delight them. The more satisfied a customer is with a brand or business, the more inclined they will be to return and recommend it to others. At the beginning of the new millennium, customer satisfaction seems to be everywhere. Customer satisfaction is a significant element in service delivery because understanding and satisfying customer needs and wants can engender increased market share from repeat purchases. The orientation to customer satisfaction is not a recent phenomenon. A number of successful business people over the years have identified the importance of customer satisfaction and output of it in a business result. Generally, customer loyalty is a behavior, while customer satisfaction is an attitude. Therefore, there are certain differences between the factors that influence customer satisfaction and customer loyalty. Price, quality, reliability, empathy, and responsiveness are the main factors that influence customer satisfaction and loyalty. Some of the factors that influence customer satisfaction and loyalty are discussed separately.

Apple may be the perfect example of a company that utilizes emotions to create a connection with consumers and brand loyalty over time. Apple's branding strategy uses simplicity, a clean design, and, most importantly, a desire to become part of a lifestyle movement. If a company fails to meet the needs of its customers, it will have a difficult time growing (Tao, 2014). If consumers buy a certain product and feel that the product benefits them, then they may get satisfied; but if the same product might not fulfill other needs, that could lead them to be dissatisfied.

**Figure 4:**
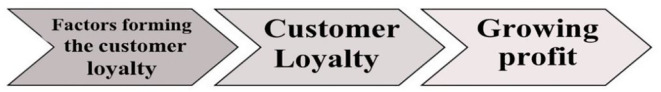
Model of customer loyalty.

Figure 4 shows how client loyalty is a process. Customers' needs and wishes are the basis of a customer loyalty model. The firm must concentrate on price, marketing, customer service, and product quality in order to build customer loyalty. Creating a comfortable atmosphere for both the consumer and the organization is crucial. The productivity and profitability of a business are enhanced when it makes an investment in a customer base of loyal customers. By understanding consumer personality, marketers can better tailor the design of websites to be most favorable for target audiences and valuable customer segments. Another important reason to understand consumer personality is because brand personality and consumer personality interact.

**Figure 5:**
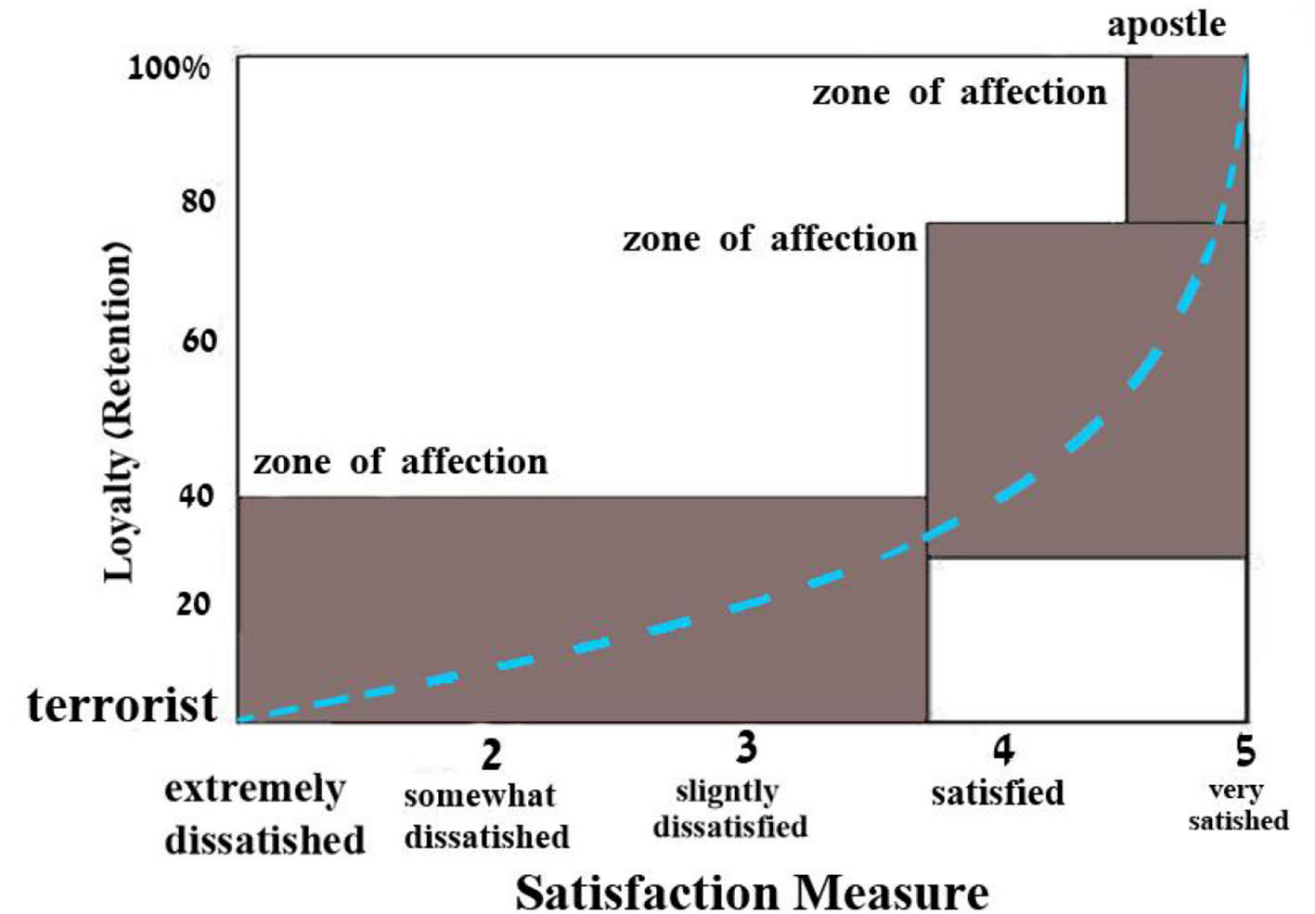
Client satisfaction and loyalty are intertwined.

The graph in Figure 5 displays client loyalty depending on satisfaction. It splits the clients into three categories and three zones. The terrorist is a “very unhappy” client. Unhappy consumers are more likely to complain about bad treatment at every chance. They may even dissuade acquaintances from trying a new service or product. “Apostles,” on the other hand, are satisfied customers who stay loyal to the company. If customers are satisfied, they are prepared to pay a premium. Every satisfied customer spreads the word to their friends and neighbors. Therefore, customer satisfaction is the primary driver of brand loyalty.

**Figure 6:**
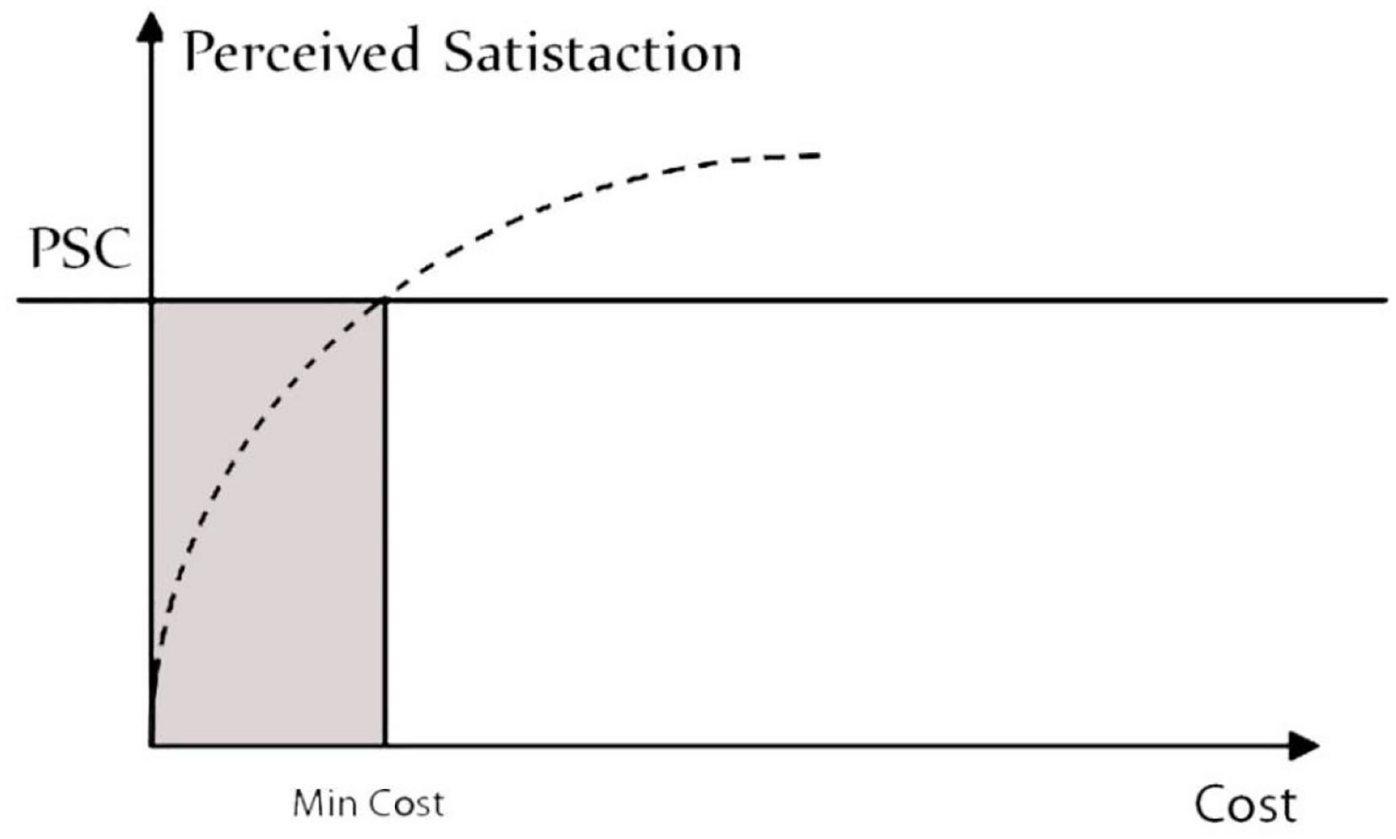
Cost–benefit link between perceived pleasure and expenses.

To assess client profitability, the link between cost (minimum) and revenue (maximum) is determined, taking into account real-world market circumstances (perceived customer happiness), representing the cost-to-customer satisfaction relationship diagrammatically (Figure 6).

**Figure 7:**
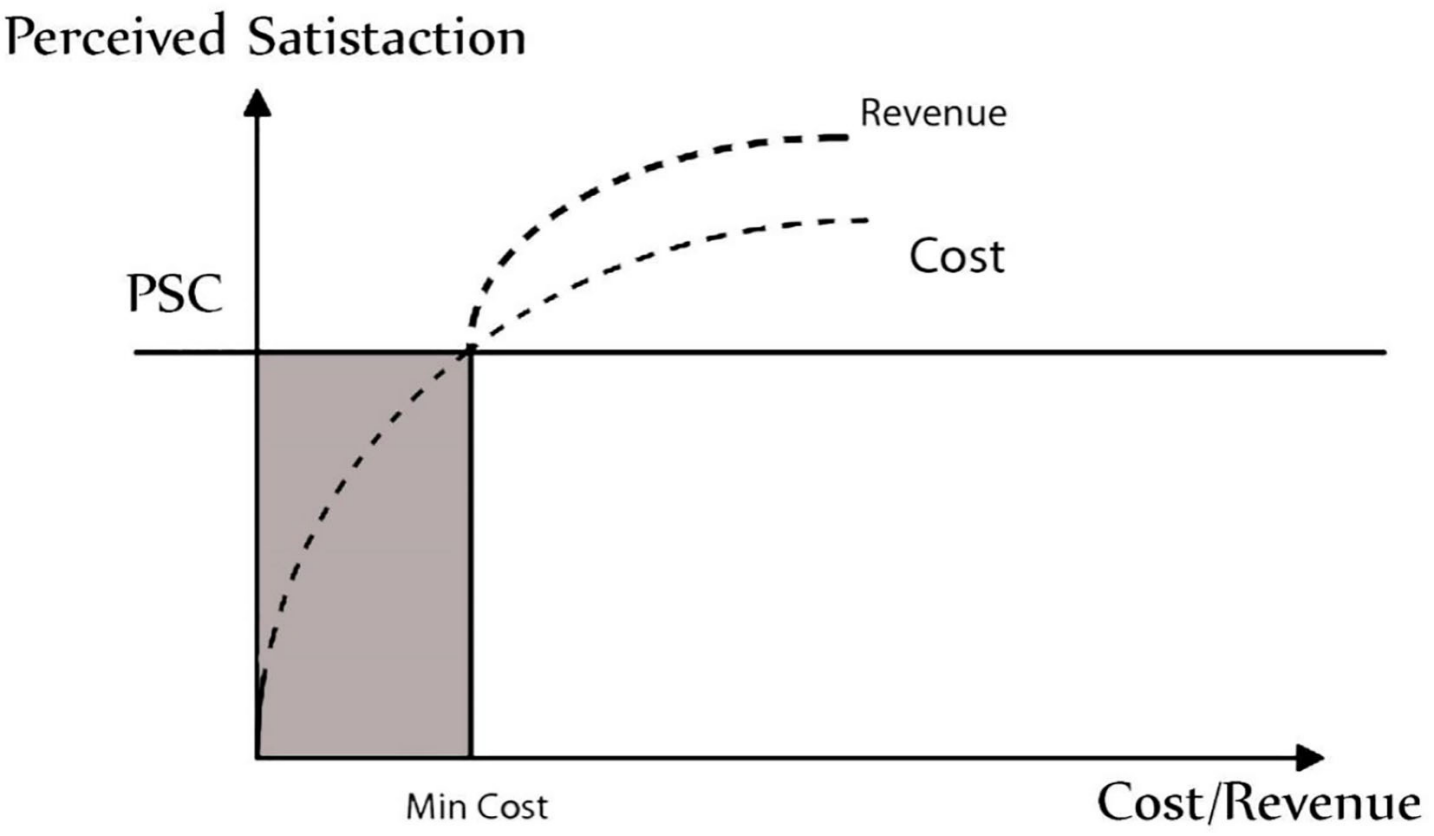
Relationship between the levels of perceived satisfaction, costs, and revenues.

**Figure 8:**
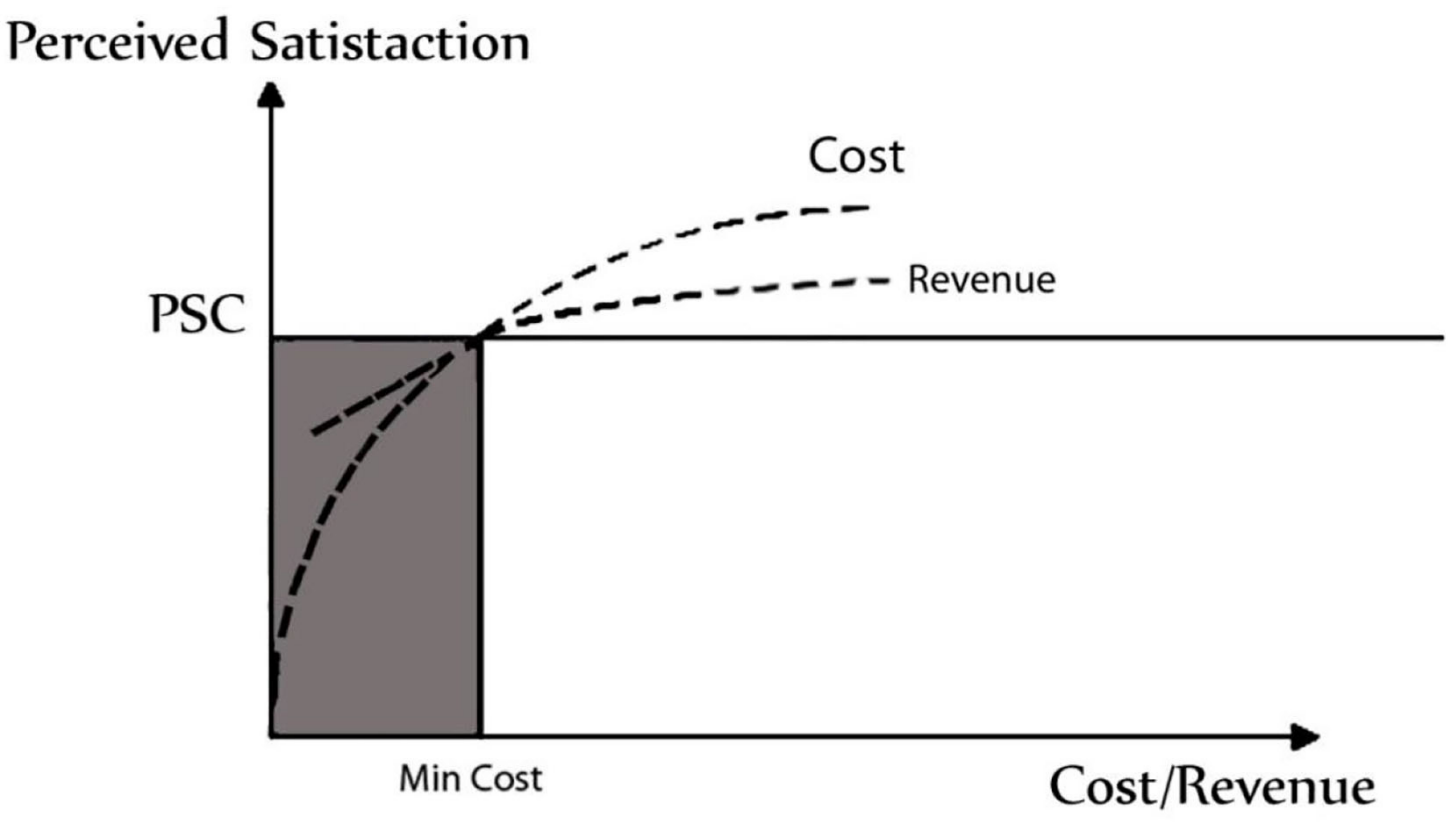
For an unprofitable section of clients, the link between perceived pleasure, expenses, and revenues.

The link between cost, revenue, and customer happiness may be graphically portrayed in order to estimate company revenues (see Figure 7). This approach may help companies estimate costs necessary to generate a certain level of revenue, as well as the profitability of a certain client group (with a specified satisfaction profile). Figure 7 is just a random example. In actuality, the graphical depiction of the cost–revenue–satisfaction connection might be rather different. For example, it is feasible that the expenses of achieving a certain level of customer satisfaction are more than the potential earnings for particular clients/consumer groups. In this case, the client/customer group would be deemed unprofitable and removed from the target market of the company (see Figure 8).

### Management expectations of customers

**Figure 9:**
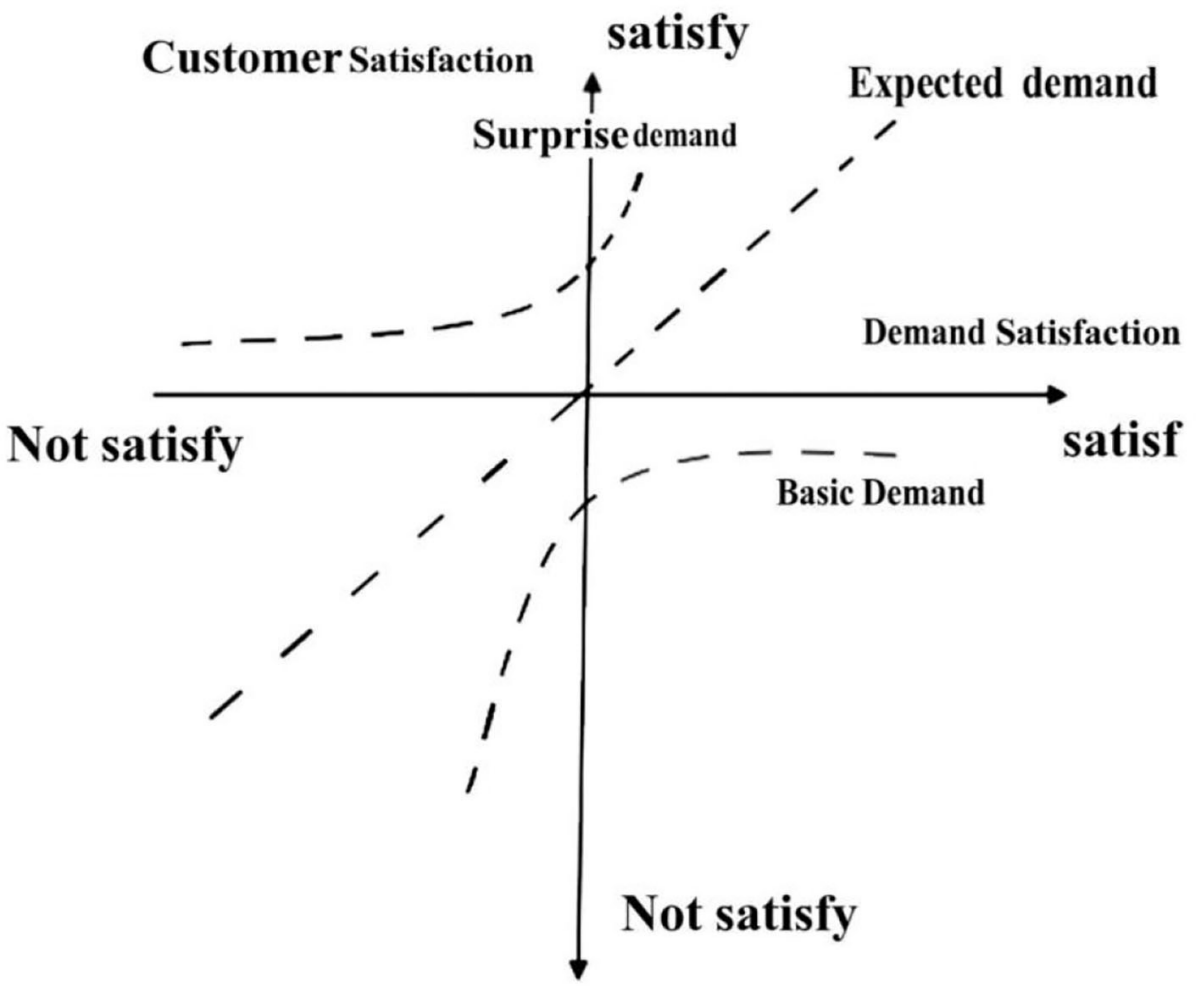
Indicate customer demand levels.

As shown in Figure 9, customers' fundamental need is considered service, and both low and high levels of satisfaction are not very happy. There is a clear correlation between expected demand and customer satisfaction and a low degree of satisfaction, while a high standard of satisfaction results in complete fulfillment, which acknowledges the clients' position in the firm's thinking.

**Figure 10:**
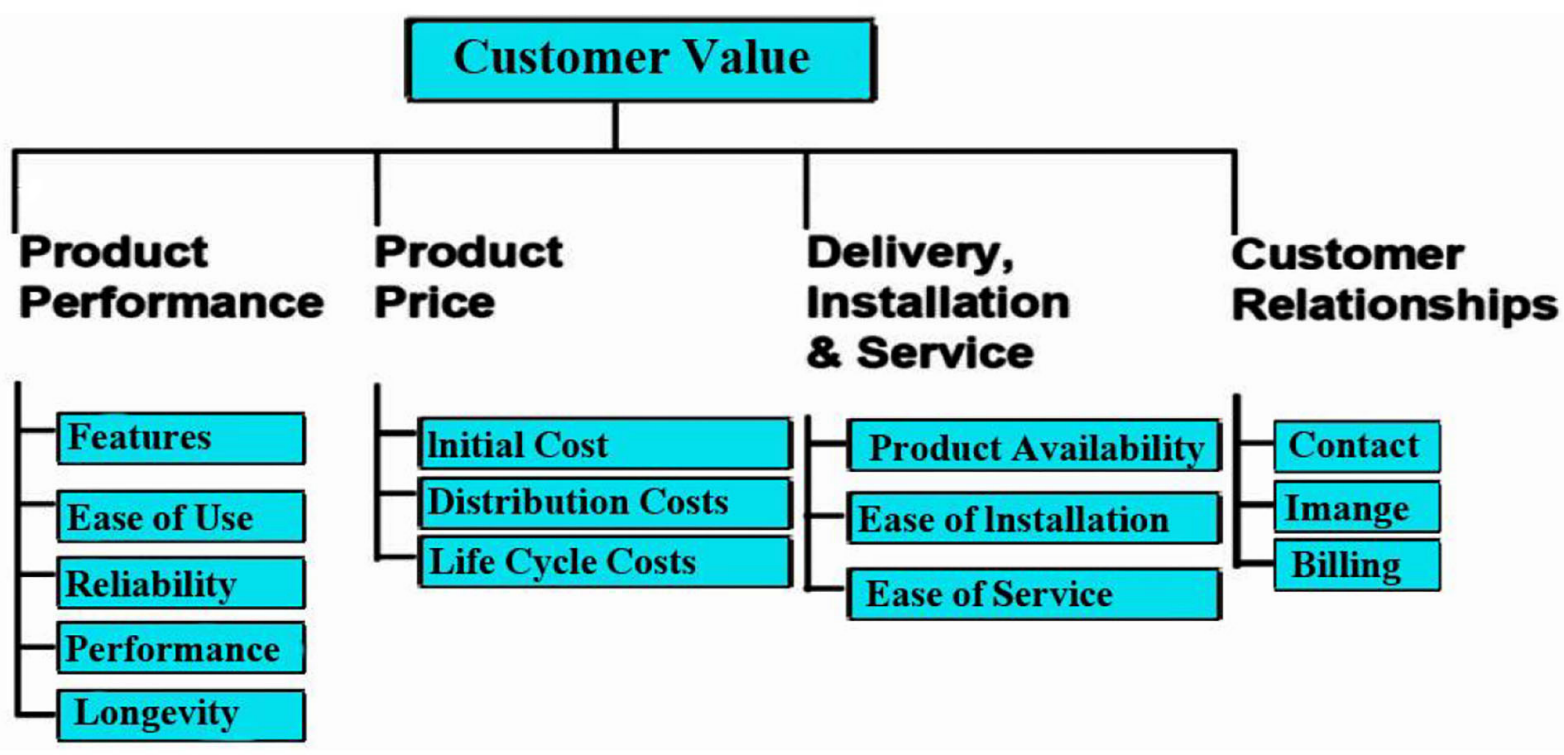
Customer value tree.

Figure 10 depicts a customer value tree, highlighting essential aspects in maintaining customers. In other words, consumers value a product or service based on their perceptions of its benefit. It should be trustworthy, durable, and feature-rich. Conversely, the product price defines the customers' importance. Customers look for products and services before paying. Valuation increases if the product price fulfills client expectations. But, along with pricing, product, etc., it is vital to preserve customer relationships. Keeping in touch often will attract clients and enhance the organization image. This demonstrates the focus of the organization on client satisfaction. The firm also makes clients feel valued, which encourages them to remain loyal customers.

**Figure 11:**
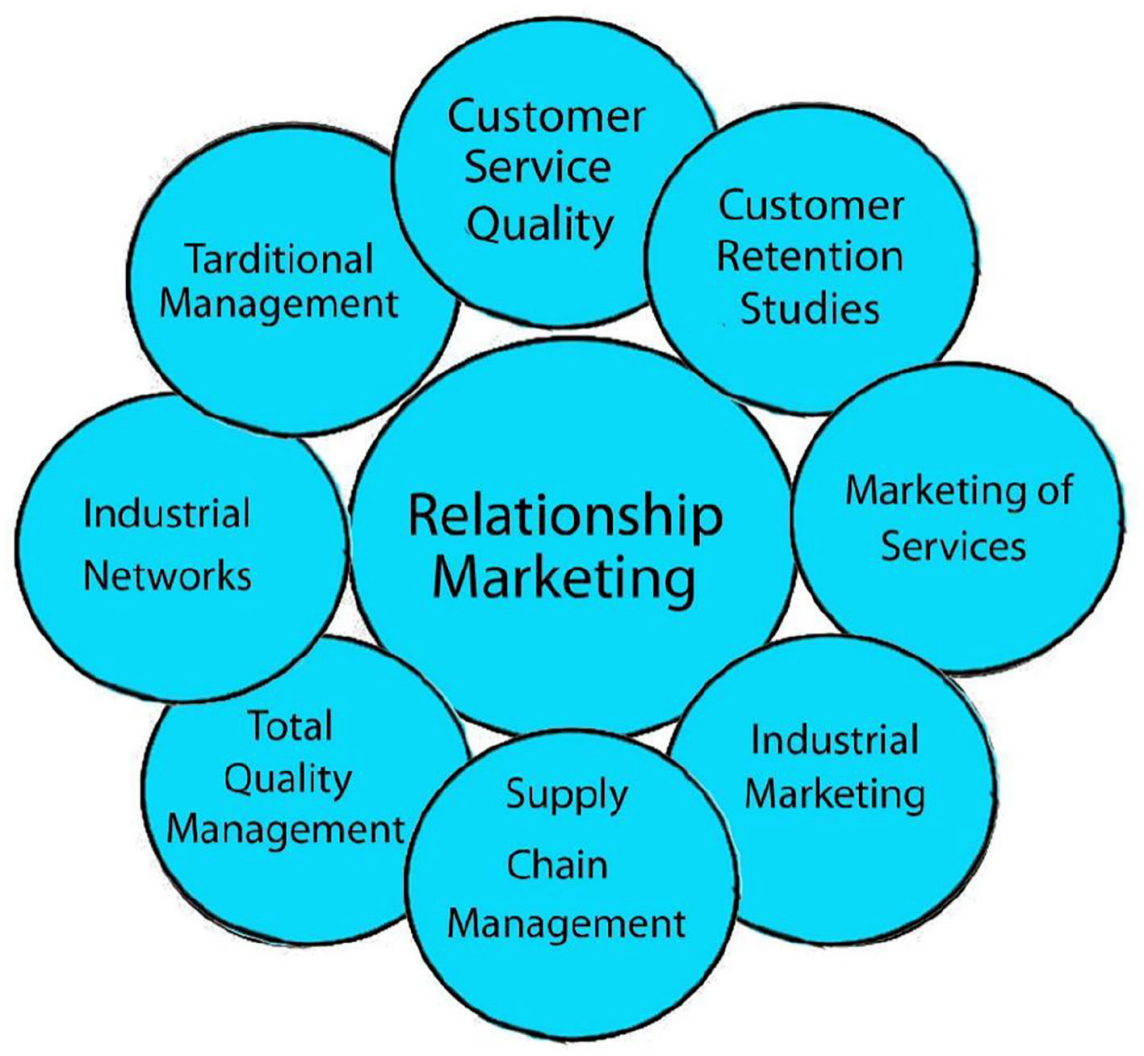
Relationship creative's primary determinants.

Figure 11 depicts the connection marketing link between the components. As shown in Figure 11, consumers and customer connection marketing rely heavily on companies. In order to build true customer connections, it is vital to keep in touch with the organization. Customers expect greater service from corporations, while firms cater to their requirements and desires. Relationship marketing requires market research. Another remarkable technique is to gather people's input. Globalization of markets and relationship marketing can boost profitability and competitive advantage.

Relationship also aids in understanding client needs. Customer retention is another factor that affects customer relationship marketing. The customer relationship marketing method focuses on retaining customers, rather than merely gaining new ones. In summary, customer relationship marketing represents a dramatic transition from bulk to customized marketing.

### SWOT analysis of Trivsel

SWOT stands for strength, weakness, opportunity, and threat. One of the most powerful tools for developing great strategies is SWOT analysis. SWOT analysis is a powerful tool for achieving goals and avoiding hazards. Advantages and limitations of a company may be found both outside and within, such as in its suppliers and competitors (Bplans, 2017).

In addition to business and industry, SWOT analysis may be used for personal and societal development. SWOT analysis aids in problem-solving creativity, as well as for adjusting and refining ideas in the middle. To achieve a goal, weaknesses must be improved, as well as the market position of the organization. Threats are prospective issues that may be evaluated. In order to make a strategic strategy or decision, a SWOT analysis must be performed (Barker et al., 2008).

**Figure 12:**
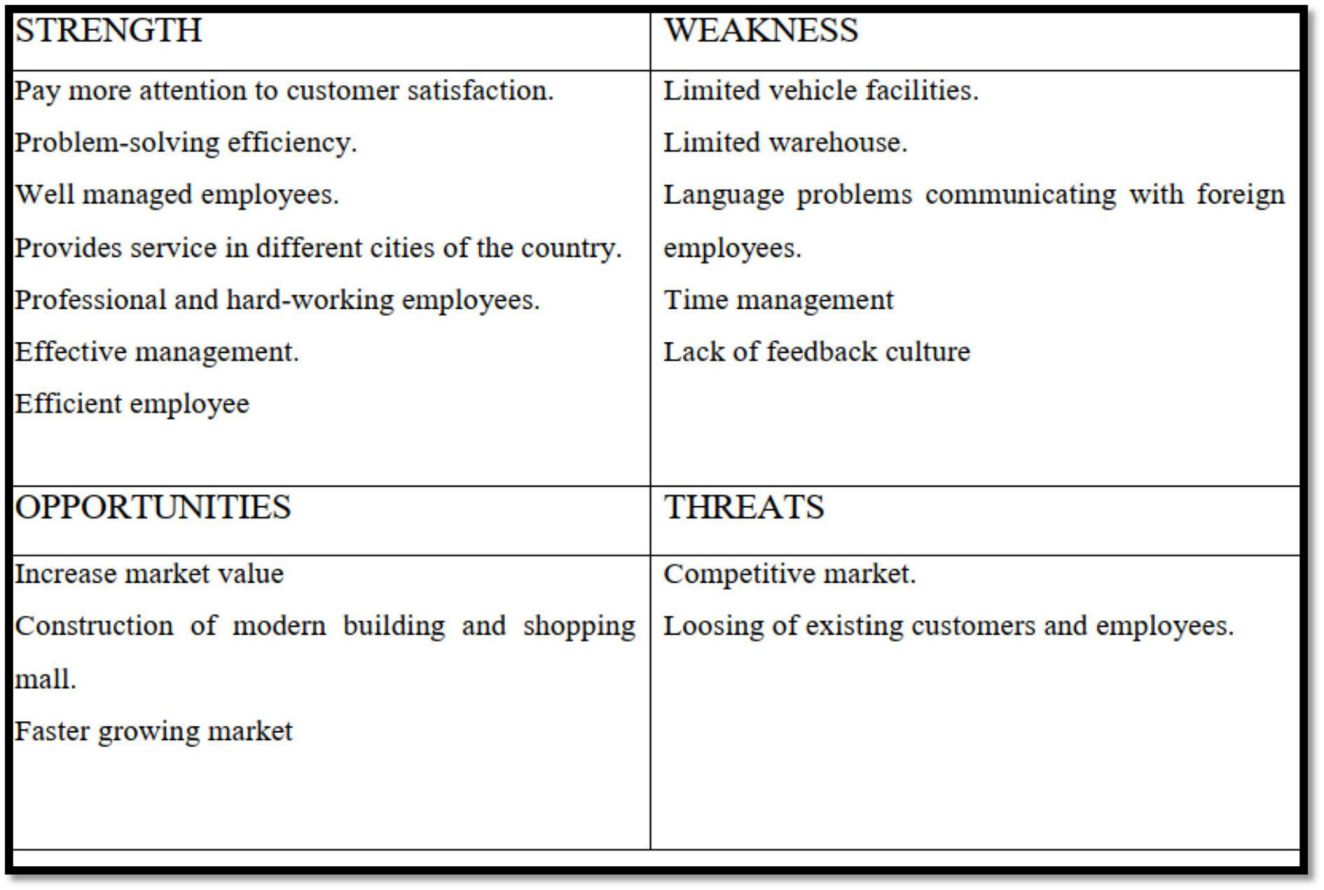
SWOT analysis of Trivsel.

Internal and external opportunities and dangers of an organization are extensively studied to develop a strategy, according to Business Dictionary, and environmental and social impact assessments. It is used to determine strengths and weaknesses of a company. Strengths, weaknesses, opportunities, and dangers of a company are the primary topics. SWOT analysis of the current study is based on the authors' own experience and an interview with Trivsel's operational director and project manager (see Figure 12).

#### What is market orientation and how does it relate to research?

Several authors have devised scales for assessing one's market orientation. Some of those assess marketing actions, while others measure goals and objectives of an organization (Deng and Dart, 1994). A market orientation is a combination of information generation, delivery, and reaction, according Kohli and Jaworski (1990). Nowadays, it is possible to separate the design and implementation of a response. For them, the concept has three behavioral components (customer focus, competitive focus, and inter-functional coordination) and multiple decision factors (long-term emphasis and a profit objective). Deng and Dart (1994) proposed a final design that has four different components, like Narver and Slater's model. This equation includes customer focus, competitive focus, inter-departmental cooperation, and profit organization.

Thus, these different market orientation constructions vary in the items they use to represent them, and there is an ethical and methodological overlap between the three of them. To answer this dilemma, it seems that there is no one meaning of market orientation. The academic literature on market orientation has offered various distinct perspectives. To put it another way, customer service is more important than production and cost, according to Konopa and Calabro (1971). The incorporation of promoting operations into the strategic decision-making process and the active participation of marketing executives are critical to firms that wish to adopt a market-oriented strategy, according to Felton (1959). Overall, three essential elements are clearly evident: consumer orientation customers, competition focus, and responsiveness are the terms used to describe the ability of a company to promptly respond to consumers' needs. A detailed analysis of the literature and consultations with 24 marketing filmmakers of machine tool companies indicate that in recent decades, manufacturing marketing has increasingly focused on customer satisfaction in order to supply high-quality goods. This shows the significance of retaining customers and establishing long-term relationships with them. Customers' long-term loyalty is critical to the success of certain firms. Businesses might observe a considerable boost in profits by keeping their customers. In order to boost profits by 10%, it is possible to retain an additional 2–5% of consumers and reduce costs by 10%. According to most researchers and practitioners, customer satisfaction is attained when the product features fulfill clients' expectations. Customers' and employees' wellbeing must be a priority for businesses if they are to meet and surpass consumer and competitive expectations. The results gathered through a structural equation model provide evidence that negative emotion experienced by consumers impacts negatively overall satisfaction, while positive emotion has a positive effect on overall satisfaction. Dissatisfaction stems from a failure to meet one's expectations. Marketing efforts that prioritize customer happiness will do all they can to obtain future orders of the company in line with the recommendations of the present pleased customers since this has a huge influence on future purchase choices. Understanding the role emotion plays in shopper behavior when they have to make a decision is crucial. Emotions from the past involving similar experience influence the choices they are considering. These emotions create preferences that lead to purchase decision. Most importantly, emotions push us toward action.

The term “market orientation” refers to a set of practices aimed at increasing customer satisfaction *via* improved performance of products (such as machines) and services related to those products (such as training and maintenance) while also maintaining a competitive advantage (pricing, delivery, responsiveness, and so on).

In this study, definitions of market orientation (Narver and Slater, 1990; Deng and Dart, 1994) are blended to develop a new business performance category that does not include profit as a focus. Since profit is the outcome of applying market orientation, we believe that profit should be considered the social component of relationship marketing.

This study contrasts the previously mentioned three market orientation constructs. As a result, we sought for any areas of convergence between these structures that would allow us to expand the market orientation domain. Finally, a group of products was constructed for aims of this study following the comparison of three separate market orientation scales. In order to select the components of the new scale with as much precision as possible, a comprehensive analysis of the area of each construction was performed. In addition to this, we considered whether or not the word was original and whether or not it had the capacity to communicate a variety of different meanings. In an attempt to reduce the amount of bias introduced by the answer set, some of the items were recoded.

### Researchers' approach to gathering data

The combination of these scales yielded 45 items as a first step. In the first place, the scope was really enormous. In light of the fact that Jaworski and Kohli and Deng and Dart are both Canadian and American scales; it was important to adapt these scales to the U.K. business culture. All five items were pre-tested by scientists and teachers in the United Kingdom to verify they were clear and appropriate for market use. We contacted professors and speakers to seek their comments on the survey. In addition, they were asked whether they would have any suggestions for improving the content or layout of the survey. Each of the 45 questions included one seven-point Likert-type scale, which allows respondents to indicate how much their organization adhered to the processes.

Rephrasing some of the themes may be necessary according to participants' suggestions. A total of two things were omitted from the list because these did not seem to be properly related to the machinery tool industry. Only after interviews were finished; 43 items, except the two, pre-test interviews were still available for use in the questionnaire provided on market orientation. After that, questionnaires were distributed to 30 U.K. machine tool manufacturers as part of the second set of pre-tests. A total of five surveys were completed and returned with only minor adjustments.

## Results and Discussion

### Data gathered from samples and analyses

The FAME-CD-ROM3 database includes 252 enterprises in Chamber Of Commerce and Industry (SIC) 3541 and SIC 3542; and 105 companies (201 companies). When the two directories were compared, 82 different companies were removed from consideration since their information was duplicated in each of the databases.

In addition, 42 businesses were excluded from the database since they were in the verge of going out of business. This left a final database with a total of 434 companies. Machine tool manufacturers in the United Kingdom received a questionnaire and an introductory letter. A confidentiality agreement was presented to each participant. They might express their interest in the study by checking the box given at the end of the questionnaire (the default choice). A move letter was sent 6 weeks later. The first and second messages consisted of a total of 93 useable evaluations, with 27 useless replies (such as the audiences having moved or the firm must be in receivership) after a 9-week period. The overall useable response rate after the first mailing was 18 points higher (73/407) than the second e–mails, which was 5% (20/334), resulting in a large response rate of 24 percent. Nonresponse bias had also been surveyed again using the tide methodology (Filion, 1975). No people are closely interrelated to the postulated final questioner in wave couple of the technique, which is relied after the first longitudinal beams of data. Others who did not respond were believed to have in the third batch of responders.

An analysis using the chi-square test showed no change in any of the variables that examined both first respondents and those in the second wave [e.g., sector of activity (i.e., CNC, non-CNC, or both), 2 = 0.79, *P* > 0.05; British or non-British enterprises, 2 = 0.46, *P* > 0.05]. The fact that the observed results did not even have an elevated nonresponse bias is embraced by previous studies as well.

A percent response accuracy was determined in a nearly equivalent poll in the United Kingdom (Pitt et al., 1996). Analysis of the probability value (VIF) revealed there were no more substantial relationship between power scalar examples (Neter and Godfrey, 1985). On the other hand, in reality, the VIF score was below 3 (Table 1 shows the features of the sample).

**Table 1:** Sample characteristics.

	**Attributes**	**Sample percentage**
Business type	British	73
	Joint venture	33
Machine category	CNC	43
	Non-CNC	33
	CNC and non-CNC	33
Earnings denominated in British pounds	Less than 10 million	68
	Between 10 and 25 million	J6
	More than 25 million	25
Employees	Less than 99	67
	From 100 to 200	26
	Greater than 200	16
Respondents	CEOs	40
	Directors serving on the board	29
	Senior	20

### Sample frame attributes

#### Market orientation

To identify the underlying features of the market orientation, we performed a factor analysis. The hypothesis of market orientation was also addressed in this study. Questions having a mean five-point level of 4.9 or above were included in the fiberglass market orientation score calculation. The addition of items with both a mean score of less than 4.9 did not significantly increase the variance, according to the component analysis. Tables 2, 3 display the data.

**Table 2:** Variance of the component analysis.

**Items**	**Mean**	**S.D**.
We make it a point to get together with clients on a yearly basis, if not more often, to discuss the goods and services they anticipate purchasing in the next years (W/JK).	5.6	1.5
People from our production department engage in direct conversation with consumers in order to have a deeper understanding of their needs and preferences (JK).	4.9	1.3
We are not very good at recognizing changes in the product preferences of our clients (R/JK).	4.7	1.2
We get knowledge on the sector *via* unofficial channels, such as having lunch with friends who work in the industry, having conversations with business partners, and so on (JK).	4.8	1.1
We are slow to recognize key trends in our sector, such as competition and technological restrictions (R/JK).	4.78	1.52
Our company's marketing staff spends time talking with representatives from different functional areas about the future requirements of our clients (JK).	4.8	1.3
When anything big occurs affecting a large customer or business, the whole business activity is notified within a short period of time (JK).	5.2	1.43
There is a minimum contact between the marketing and production departments concerning market development (R/JK).	5.1	1.6
There is a lag in the rate at which departments share information about competitors with one another (W/R/JK).	5.3	1.23
We have a propensity to neglect changes in the requirements for the products or services our clients need (R/JK), and the reasons for this vary.	5.9	1.15
We perform frequent inspections of our item development efforts to assure that they are matched to our customers' demands and tastes (JK).	5.4	1.7

**Table 3:** Variance of the another component analysis.

**Items**	**Mean**	**S.D**.
In general, our response time to rival marketing initiatives directed toward our client base (W/JK) is quite swift.	4.92	1.33
The several departments that make up this business unit's operations are working together effectively, as a result (JK).	5.13	1.24
Complaints from clients are not taken seriously by this division of the company (R/JK).		
Even if we devised an excellent strategy for marketing our products or services, there is a good chance that we would be unable to put it into action in a timely manner (R/JK).	5.75	1.51
When we learn that consumers are dissatisfied with the quality of the services we provide, we quickly take steps to address their concerns (JK).	5.13	1.54
When we discover that consumers would want us to make changes to a product or service, the relevant departments immediately begin working together to implement those changes (JK).	5.9	1.3
There is not much of a difference between “sales” and “marketing” (W/DD) at our firm.	5.92	1.2
The most essential function that marketing performs for our organization is to advertise our goods and services to our clientele (DD).	5.2	1.6
The most essential function that marketing does for our organization is determining the requirements of our clients and working toward their satisfaction (DD).	5.3	1.21
In order for the firm to achieve a competitive edge (W/NS), it focuses on pursuing certain possibilities.	5.4	1.15
Within our company, each department contributes in some way to the production of value for the client (W/NS).	5.34	1.19
Within our company, the individuals who work in marketing have regular interactions with employees working in other areas, such as production, finance, distribution, and so on (DD).	5.43	1.53
Within our company, the individuals in charge of marketing have significant impact on the process of developing new goods (W/DD).	5.16	1.48

Orientation toward consumers (F1) and orientation toward competition (F2) were the two different criteria identified (F2), which is another aspect related to responsiveness, which is in line with the notion. Responsiveness was so named after the factor (F3). The fourth aspect is focused on customer service (F4). In this case, four factors explained roughly 67% of the variation. Table 4 summarizes the variables' descriptive statistics and dependability.

**Table 4:** Variables' descriptive statistics and dependability.

**Item no**.	**Fl**		**F2**	**F3**	**F4**
19		0.79			
19		0.77			
13		0.72			
22		0.66			
22		0.65			
10		0.63			
9		0.59			
1 2		0.58			
24		0.57			
3		0.53			
15		0.52			
17		0.51			
5			0.66		
17			0.66		
23			0.64		
24			0.53		
16			0.52		

### Performance measurement

There were five different ways to measure how well a company was doing. All parts of this design include customers (P CUSTRET), share of the market (P MKTSH), sales growth (P NPS), ROI, and growth in sales (P SG). On a seven-point Likert-type scale, 1 means strongly disagree and 7 means strongly agree. Performance may be evaluated based on a person's financial or organizational benefits. In the questionnaire, questions were included about the long-term viability of a company's profits. Respondents rated their personal performance over the last 3 years to that of their peers. Drivers are more likely to provide accurate 3-year profit estimates. Since firms are increasingly financing expensive equipment only through the sales force of micro-finance institutions after such a contract has been signed, a 3-year time frame provides an indication of corporate profitability. Over the last 3 years, questions were required to assess their satisfaction with the machine, which serves as a proxy for customer retention. Respondents may be more likely to be affected by the excellent efficiency of a new (1-year-old) computer than even an old system (which might be impacted by the recency effect) if the time period was set to 3 years (3-year-old). Using a subjective point of view was necessary due to the difficulty in collecting objective data from documented sources. Because companies were unwilling to provide material that may be considered confidential, it was hard to conduct an investigation with objectivity. We all know how tightly objective measurements and individual responses are intertwined.

A single factor solution was obtained using principal component analysis (eigenvalue >1). Table 5 shows the findings of the study. The five-item measure has a Cronbach's alpha of 0.88 and a standardized Cronbach's alpha of 0.87, respectively.

**Table 5:** Reliability examination of business performance.

**Performance indicators**	**ltem-to-item correlation**	***a* if item deleted**
P_CUSTREN	0.48	0.88
P-MKTSHR	0.75	0.84
P_NPS	0.51	0.88
P_ROI	0.72	0.85
P_SG	0.76	0.84
Cronbach o = 0.87	Standardized Cronbach o = 0.86	
Eigenvalue = 3.85	Variance explained (factor analysis) = 63.5%	
Factor mean (BP) = 5.46	BP factor standard deviation 6 = 1.18	

The scale scores were satisfactory and above the indicated cutoff threshold of 0.70 for the scale (Cronbach and Webb, 1975; Nunnally, 1978). Another point in favor of the reliability of the measure is that the variation between alpha and standardized alpha is negligible (which accounts for scale length). The factor representing company success was the mean raw score of these five elements. The investigation sought to identify key variables explaining variation in company performance. The regression model explained 37% of the variation in company performance, according to the findings (Table 6).

**Table 6:** F = performance in business (parameters in market orientation).

**Factors**	***Fl***	**S.E. (€)**	***t-*value**	***P-*value**
FI (customer orientation)	0.28	0.07	2.72	0.04
F2 (competitor orientation)	0.22	0.14	2.43	0.06
F3 (responsiveness)	0.03	0.06	0.18	0.78
F4 (customer satisfaction)	0.23	0.08	2.54	0.03
li' = 0.39	F = 9.16			
Adjusted R' = 0.37	11 = 94			

**Figure 13:**
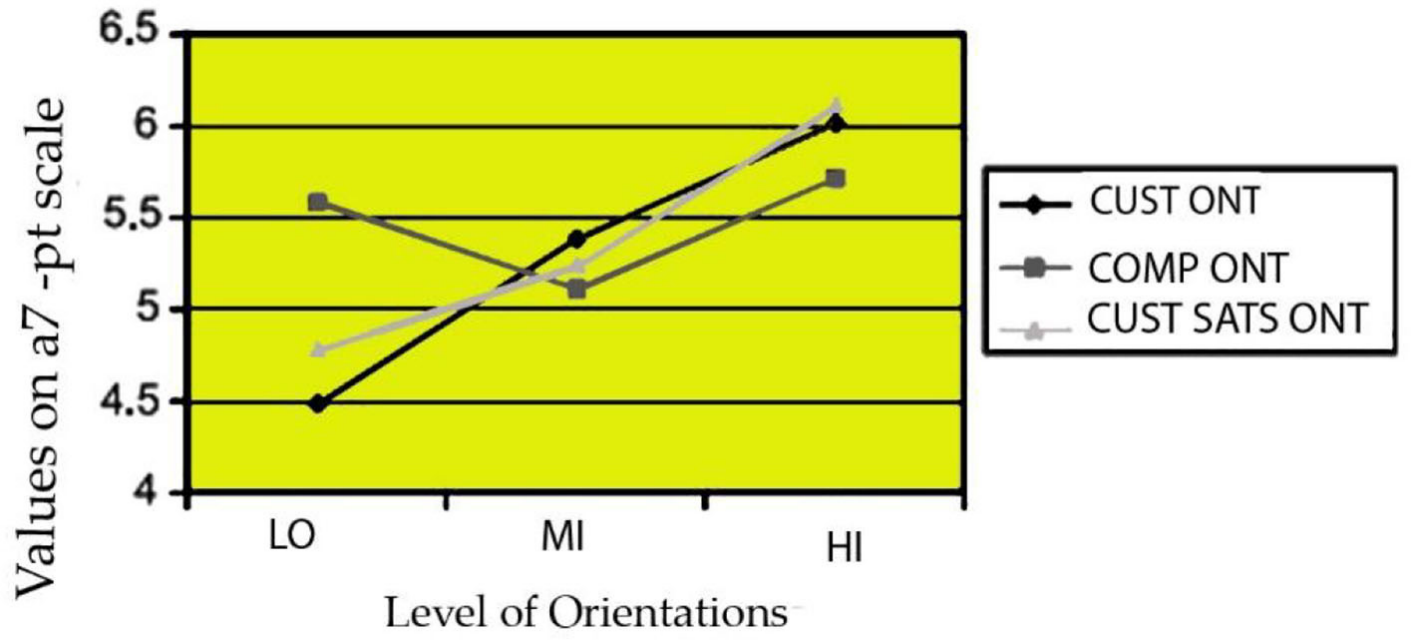
Relationship between corporate performance and degree of orientation.

For testing the uniformity of average scores across groups, subgroup analysis seemed appropriate since multivariate regression analysis is useful for analyzing the connection between a predictor variables and numerous independent factors. All components were classified into low, medium, and high (LO to MI and H to H) subgroups (HI). For each component, cutoff values were determined to ensure that each group received about the same number of replies. Table 7 shows one-way ANOVA findings. Subgroup mean values are shown in Figure 13 for visual comparison.

**Table 7:** Analysis of variance (ANOVA), the average of the strategic marketing factors.

**Factors**	**FI (CUST orientation)**	**F2 (COMP orientation)**	**F'4 (CUST satisfaction)**
LO	4.48	5.57	4.79^*^
MI	5.37^*^	5.12	5.23^*^
HI	6.03^*^	5.72	6.12^*^

* Significant at P < 0.06 level.

## Conclusion

The objectives of this study were to develop a concise industry-specific entrepreneurial orientation scale and to investigate the underlying factors that represented the market orientation concept. According to the data from this sample, there are four major dimensions, three of which are significant. These factors were given customer orientation, competitive orientation, flexibility, and patient satisfaction orientation. Regression analysis was used to assess the impact of each individual orientation on firm performance. The findings confirm our premise that customer orientation, competitive orientation, responsiveness, and user satisfaction orientation are critical factors influencing corporate performance. However, it was not shown that departmental responsiveness was significantly and positively connected with corporate performance. This is a surprising result, since one would expect immediacy to be a major determinant in consumer happiness. Companies that are happy with broad customer-oriented methods may take departmental reaction for granted. Corporate performance is improving, according to ANOVA. ANOVA revealed that organizations perform better when they are more customer-competitive and customer satisfaction-oriented by effectively integrating activities across several divisions within a company.

### Limitation and future research

As with most research efforts, this study has limitations too. One of the limitations of the research is that respondents were asked to score subjectively on a seven-point Likert-type scale. These evaluations are subject to personal bias and judgmental errors. However, financial constraints necessitated us to use this methodology. Future research could include a multiple-respondent methodology and use objective data from company reports to ascertain financial performance. It would also have been useful to measure the extent to which market orientation strategies contributed to repeat purchases. Furthermore, customer satisfaction could be measured as the percentage sales from repeat buying. It is important to mention that the study provides only a snapshot picture at a single point in time, which means that the recommendations are valid only if external environmental variables are unaffected, for example, government regulations, foreign exchange, economic cycle, and competitiveness of the developing nations to produce these machines at a lower cost. It will be interesting to know if these variables moderate the relationship between various dimensions of market orientation and business performance. It is also desirable to develop a model using LISREL to detect the causal effect of these dimensions on performance. The modest sample size places limitations on the confidence in our findings. Repetition of the study with a bigger sample would help validate the findings as we have not found responsiveness to be significantly related to the business performance. Nonetheless, the findings of the consequences of market orientation on performance do shed some light on the understanding of the impact of market-oriented activities. We hope that our study warrants serious consideration to practicing managers about how customers, competitors, and customer satisfaction focus can contribute to enhancing performance of their companies in the short and long terms in light of external environmental variables. Finally, the findings offer an insight into the machine tool industry but fall somewhat short of full generalizations. However, the industry-specific construct could be used as a test bed for further research into other manufacturing industry sectors in other countries.

## Data availability statement

The original contributions presented in the study are included in the article/supplementary material, further inquiries can be directed to the corresponding author.

## Author contributions

LZ: writing and static analysis of data. YG: guiding the research directions and ideas. WC and HR: experimental operation and check. All authors contributed to the article and approved the submitted version.

## Funding

This work was supported by the Xi'an Social Science Foundation (22JX108), Xi'an Eurasia University Technological Service Special Program (OYJSFW-2021001), and Shaanxi Social Science Foundation (2020D018).

## Conflict of interest

The authors declare that the research was conducted in the absence of any commercial or financial relationships that could be construed as a potential conflict of interest.

## Publisher's note

All claims expressed in this article are solely those of the authors and do not necessarily represent those of their affiliated organizations, or those of the publisher, the editors and the reviewers. Any product that may be evaluated in this article, or claim that may be made by its manufacturer, is not guaranteed or endorsed by the publisher.
